# American Academy of Dental Science

**Published:** 1894-06

**Authors:** 

**Affiliations:** American Academy of Dental Science


### AMERICAN ACADEMY OF DENTAL SCIENCE.

The regular monthly meeting of the American Academy of Dental Science was held at Young's Hotel, Boston, on Wednesday evening, February 7, 1894, at six o'clock, President Smith in the chair.

The paper of the evening was read by Dr. H. E. Cutter, of Cambridge; subject, "A Case of drawing the Lower Jaw Forward;" illustrated by models.

(For Dr. Cutter's paper, see page 353.)

### DISCUSSION.

*President Smith.*—Gentlemen, this very interesting case of regulating, which has been so nicely presented by Dr. Cutter, is before you for discussion, and we shall be glad to hear the remarks of any members upon the subject as the models are being passed about.

*Dr. Fillebrown.*—At what age was this commenced?

*Dr. Cutter.*—The patient was eleven years of age.

*President Smith.*—The plates were all removable at the will of the patient?

*Dr. Cutter.*—Yes, and the patient experienced no marked inconvenience.

*Dr. Bradley.*—And when was it completed?

*Dr. Cutter.*—When the patient was twelve years of age. The work extended over the time when the twelfth-year molars were erupting.

*Dr. Fillebrown.*—Are all the plates off now?

*Dr. Cutter.*—Yes; and I have tried the patient a number of times, and cannot get her to bite in any other than her present way.

*Dr. Bradley.*—May I inquire if this plate was split at all?

*Dr. Cutter.*—It was sawed through the middle. Sometimes I cut a plate in two places and swing one or both sides on a pivot which is inserted after the plate is vulcanized.

*Dr. Bradley.*—I don't think I understand how the lower jaw was moved forward. A plate was first made by which the lower incisors were prevented from striking on the gum; immediately after that what was the next step?

*Dr. Cutter.*—The platform plate, which kept the lower incisors from touching the gum, at the same time caused them to go forward a little.

*Dr. Bradley.*—The movement was not on the bicuspids and molars so much as on the incisors?

*Dr. Cutter.*—That was the point.

*Dr. Brackett.*—Did the wearing of this appliance at the time the emphasis of the bite was upon the lower incisors produce shortening of these incisors?

*Dr. Cutter.*—I think not.

*Dr. Brackett.*—The change in the relative length of the teeth was in the elongating of the others?

*Dr. Cutter.*—Yes.

*Dr. Brackett.*—With reference to the material of the bands to go about the teeth, the essayist made some remarks to the effect that he had used gold. Some one has said that he has had satisfactory experience in the use of German silver; and I understand that anything one may require in this metal can be obtained at Harwood Bros., dealers in watchmakers' supplies, on Washington Street, Boston.

*Dr. Cutter.*—I had to be very careful not to allow the twelfth-year molars to erupt too far. If the plate had been made too thick behind the upper incisors, the twelfth-year molars would have grown up so far as to have prevented the upper and lower incisors from meeting.

*Dr. Wilson.*—How long has this been completed?

*Dr. Cutter.*—Since last summer.

*Dr. Wilson.*—Is the patient still wearing a retaining plate?

*Dr. Cutter.*—No, I had her wear a retaining plate for a long time, simply to hold the flattened position of the front teeth. I saw her quite often and watched the case very closely, and, after making many careful tests, I could not see any necessity for wearing the plate longer. Ordinarily I require a patient to wear a skeleton retaining plate for a year or more, but this case did not require it.

*Dr. Williams.*—I would like to ask Dr. Cutter if this patient apparently had any hereditary tendency?

*Dr. Cutter.*—I think she inherited the tendency from her father.

*Dr. Wilson.*—The upper plate was held in place by the clasps, was it not?

*Dr. Cutter.*—Yes, it was.

*President Smith.*—An interesting point in this connection is to determine just what took place in the forward movement of the lower jaw. Was this movement brought about by the moving of the condyle in the glenoid cavity, or was there a change in the jaw substance itself? Perhaps Dr. Cutter has made some study of that point, and can tell what, in his opinion, took place.

*Dr. Cutter.*—I have thought of that a number of times, and my own feeling was that the condyle moved forward in the glenoid cavity.

*Dr. Allen.*—May I ask if the bicuspids jumped the bite?

*Dr. Cutter.*—Yes, all the teeth jumped forward.

*Dr. Allen.*—I think that in itself is evidence that the change occurred in the angle of the jaw.

*President Smith.*—It has been held by some that the rami of the jaw were bent in such cases. I don't believe that can happen at that age; I would like to ask the opinion of some of those wiser than myself on that point. What do you think, Dr. Andrews?

*Dr. Andrews.*—I believe, if it can be done at all, it is done when the child is young. During the last year I have had three cases where I have been successful in jumping the bite.

*President Smith.*—Do I understand from Dr. Andrews that he thinks the rami of the jaw can be bent to allow of the jumping of the bite?

*Dr. Andrews.*—I think it may be possible at an early age; I have never studied the matter enough to speak positively about it.

*Dr. Stevens.*—One thing occurred to me, Mr. President, in regard to applying the force to the incisor teeth; that is, why were not the incisor teeth moved forward instead of the jaw? It seems to me it would be natural for them to follow the inclined plane.

*Dr. Cutter.*—They all move forward as one body.

*Dr. Stevens.*—Did you put any bands around them to prevent their being drawn forward?

*Dr. Cutter.*—No.

*Dr. Wilson.*—I did not understand that the teeth struck against an inclined plane, but simply against depressions in what is called a platform plate.

*Dr. Cutter.*—The plate was sloped a little, so as to guide the teeth into these depressions.

*Dr. Adams.*—I suppose the teeth were compelled to throw the jaw forward in order to get a comfortable bite.

*Dr. Cutter.*—Yes, and I am surprised how readily they will throw the jaw forward, if you do not attempt too much at a time.

*Dr. Andrews.*—I had a patient a little over twelve years of age, the cutting-edge of whose lower incisors touched the upper gum, so as to irritate it. A platform plate such as Dr. Cutter describes was worn for about two months. The lower centrals, laterals, and cuspids struck against that plate and allowed the molars and bicuspids to elongate. After a time I found there was one-eighth to a quarter of an inch space between the lower incisors and the upper gum on closing the mouth. The lower jaw was found, however, to have been moved a little to one side, and an unnatural occlusion formed. Another plate, having depressions adapted to correct this bite, was worn for about two weeks and the articulation brought into proper shape.

*Dr. Fillebrown.*—I would like to ask Dr. Cutter how many upper plates he made with depressions advancing forward?

*Dr. Cutter.*—I should say three or four. I could gain only a small amount with each plate.

*Dr. Fillebrown.*—How long did it take you to gain the first jump?

*Dr. Cutter.*—I think it was about a month.

*Dr. Allen.*—Will Dr. Cutter tell us whether the patient had much forward and back mobility of the lower jaw?

*Dr. Cutter.*—About the average amount.

*Dr. Allen.*—Not as much as you finally gained?

*Dr. Cutter.*—No. It would have been impossible for her at first to throw her jaw out as far as she was able to after the regulation.

*Dr. Allen.*—It would be interesting to know just where the change occurred, whether in the movement of all the teeth or at the angle of the jaw.

*Dr. Cutter.*—I think it was at the condyles of the jaw.

*Dr. Wilson.*—I don't quite understand how you arrange the clasps on your plate.

*Dr. Cutter.*—I carry the clasp-wires from the plate between the second bicuspids and sixth-year molars. The clasps were placed in front of the sixth-year molars, so as not to interfere with the eruption of the twelfth-year molars.

*Dr. Bradley.*—Did the patient wear this platform plate when eating?

*Dr. Cutter.*—I think not. I told her that she might take it out during meal-time, but must put it back immediately afterwards. I impress upon patients the necessity of keeping their plates, as well as their teeth, clean during the process of regulation. The teeth of this patient were not particularly strong, yet she brushed her plates so well that I saw no injurious effects from their use.

*President Smith.*—I am surprised that Dr. Cutter allowed his patient to remove the plate while eating. The appliance was arranged to produce a change in the manner of closing the teeth, and yet the patient was allowed to take it out when she ate, and that is the only time, commonly speaking, when we put our two jaws together.

*Dr. Fillebrown.*—It is not alone when we eat that we put our jaws together.

*President Smith.*—It is safe to say that, as a rule, the teeth stand slightly apart, and for that reason I should think it quite important in Dr. Cutter's case that the appliance should be worn while eating.

*Dr. Stevens.*—I would like to ask the President if he ever had for a week or two a very sore tooth in his mouth?

*President Smith.*—No, I never did, except a little soreness from wedging?

*Dr. Stevens.*—I think if you had had such a tooth, you would have found that you closed your teeth very often during the day.

*Dr. Williams.*—Did Dr. Cutter give his patient any instructions with regard to the method of biting when not eating?

*Dr. Cutter.*—I instructed her to avoid as much as possible closing her teeth in the old way. I cannot say positively that she took her plates out while eating, but I commonly allow patients to remove regulating appliances during meal-time, on condition that they replace them immediately afterwards.

*Dr. Fillebrown.*—The gentleman at my side has reminded me that we swallow many quarts of saliva daily. The teeth naturally close every time we swallow. If you divide the quarts by the approximate amount of each swallow, you can tell how many times a day your teeth are closed for swallowing purposes alone.

*Dr. Allen.*—I am not inclined to accept the theory that the angle of the jaw was changed in this case. Supposing the mobility of the jaw to be so slight as to prevent the patient from moving the jaw forward so as to bring the upper and lower teeth into the relation shown in the models of the corrected case, the change could have been effected, it seems to me, by the very process employed by Dr. Cutter. This would have a natural tendency to stretch the muscles sufficiently to bring the jaw forward the required distance, which, as the models show, is less than the width of a bicuspid tooth. My impression is that the plate used in this case induces a new habit of closing the teeth, due to its effect upon the muscles rather than upon the bones. This was accompanied by a slight change in the position of the bicuspids and molars of both jaws. I have not found that any difficulty arises from the patient's not wearing a plate during meals, for the jaws receive much exercise in the unconscious moments of swallowing. Professor Austin Flint tells us that we swallow from four to six gallons of saliva during twenty-four hours, and as the jaws are being continually brought together in this process, the regulating goes on, despite the interruptions of eating without the plate.

*Dr. H. A. Baker.*—I would like to ask Dr. Cutter if the jack-screw which he uses in this case is an invention of his own, and if he vulcanizes it into the plate with the screw intact, and of what material it is made?

*Dr. Cutter.*—The jack-screw is made of German silver, and is vulcanized into the plate with the screw intact, but the screw can be freely turned when the plate comes out of the vulcanizer. This jack-screw is the result of certain experiments made by Dr. Andrews and myself.

*Dr. Eames.*—I understood Dr. Cutter to say that the bite was not open when he began the correction of this case; the incisors were in contact with the gum or nearly so.

*Dr. Cutter.*—The points of the lower incisors touched the gum behind the upper incisors.

*Dr. Eames.*—I do not see why in such a case it is necessary to bend the jaw at its angle in order to jump the bite. The moving of the whole jaw forward is all that is required.

*President Smith.*—Our next subject for the evening is the "Artistic Arrangement of Artificial Dentures;" it will be presented by F. S. Belyea, D.D.S., of Brookline.

*Dr. Belyea.*—I have a few drawings which I wish to show. I have tried to find suitable sketches to illustrate my points, but failing to find them, I went to work and made these myself, which I will show to you to-night. These sketches illustrate my motive for using certain mechanical contrivances, which I shall endeavor to describe. My subject naturally divides itself into two parts,—first, the restoration of facial expression; second, the restoration of the teeth themselves. In this diagram I have tried to show the features of a youth of twenty years, and in the next one I have tried to show the same person at the age of sixty, after the loss of all the teeth. In the first picture we find an elevation at the corners of the mouth, and a fulness about the mouth and cheeks. In the second there is a drooping at the corners of the mouth and a general falling in of the cheeks. This change is caused by the absorption of the alveolar processes and a relaxation of the facial muscles through the removal of their natural support. In order to restore the lost fulness, I apply plumpers in the region of the cuspid roots. To a certain extent they draw up the corners of the mouth, and then, by the application of plumpers at the side, the looseness in the buccal region is filled out. These plumpers are held in place by the muscles. I try to make my plate restore the features, and, in order to do so, I have an outline in wax, which represents the position of the teeth, gives the length, fulness, size, and shape of the arch. Some hold that only one arch should be fitted in every mouth. I do not believe in that theory at all. In getting up the articulation, you must have some idea of the length and position of the end of the teeth. I have noticed failure in many cases of artificial teeth through ignorance or neglect of this rule. If you can see this red line, that is the point from which I try to secure an occlusion. I draw an imaginary line from just below the lobe of the ear to the edge of the central, and in cutting my wax I follow that line and construct my plate accordingly. That principle was set forth in a paper printed some years ago in the *Dental Cosmos.* I think the title of it was "The Artists in Dentistry."

*Dr. Fillebrown.*—I feel that there is a limit to what can be done in restoring the parts to their former condition after the loss of the teeth. When the canine teeth are lost, the pillars of the arch are gone, and when the process has wasted away, as it will, there must be a depression at the side of the nose, which can never be restored. A person at sixty, even if no teeth are lost, will not have the fulness of cheek he had at twenty, consequently it is not reasonable to expect to restore to a person of sixty the plumpness and fulness he had at twenty by the use of any artificial appliance. The fulness of the cheek does not to any considerable extent normally depend upon the presence or pressure of the side teeth, but upon the length of the occluding teeth. A person's cheek falls in not because teeth fail to press the muscles outward, but because the shortened occlusion slackens the muscles, and the cheek falls inward; when the masseter muscle is again put upon the stretch, the cheek is restored to its normal condition. Hence I think plumpers on the side are not ordinarily required, and do not have much to do with restoring the face to the condition which it would have obtained had not the natural teeth been lost.

*Dr. Brackett.*—In behalf of the last speaker, I would say that he has made all these remarks without seeing Dr. Belyea's models.

*Dr. Belyea.*—I must beg to differ most decidedly with the gentleman when he says that plumpers have nothing whatever to do with the holding out of the cheek. I have found that they have an important effect. Of course, I do not pretend to get back to a face of twenty; I try to get it as near nature as I can, and I never have gotten too near to it yet. Simply opening the bite will perhaps allow the muscles to change a little bit, but they will not draw back this objectionable fulness to any great extent.

*Dr. Fillebrown.*—I did not intend my remarks as criticism of what has been placed before us this evening, but only of the general principle. In years past I have sometimes failed to consider what the normal condition was at sixty years of age, and how many of the lines that I sought to remove were legitimate and should remain. In our endeavor to correct the sunken appearance, we are, I think, inclined oftentimes to overdo it.

*Dr. Bradley.*—I scarcely grasped the idea of Dr. Belyea from the drawings and from his remarks, but seeing the models which have been passed around, I think I can understand it more satisfactorily to myself.

I do not do a great deal of plate-work, but I happen to be working at the present time on a plate for a lady nearly sixty years of age, who lost her teeth through pyorrhœa. The case presented somewhat peculiar conditions, and I worked very faithfully, trying to make a satisfactory plate, and, although the patient wears the plate, yet when she attempts to bite anything off at the front of the mouth, the upper plate will loosen at the back. There is no "ridge," I might say, at the back of the jaw. I am now studying this case over again, and the thought has come to me in looking at this model,—would it be possible to give the impression of fulness which is so desirable by the use of plumpers in the cheek? The face seemed to be in a collapsed state, as it were; there was no natural expression whatever, and it occurs to me that this may help to a solution of the case in filling it out by means of plumpers. I think I shall try it.

*Dr. Belyea.*—Speaking about plumpers in the lower jaw, I have found, in the number of cases that I have had to deal with, that fulness is of not much use, or even allowable, excepting in the bicuspid and molar region. No attempt that I have made to restore fulness lost by absorption in the lower cuspid region has made any very perceptible improvement in the facial expression.

I would say that I never put in a set of teeth for anybody that I do not grind them more or less, even in young people. It adds greatly to the natural appearance of the case.

This case of removable bridge-work that I show is made by the use of Logan crowns. The plate is struck up, the pins are cut off the crowns, and they are placed on, and then solder flowed in between.

This other case is to show a bridge made so that you can set the bridge first, and then the individual crowns afterwards.

WILLIAM H. POTTER, D.M.D.,
*Editor American Academy of Dental Science.*

